# The influence of ideological and political education identification on college students' sense of social responsibility

**DOI:** 10.3389/fpsyg.2026.1836738

**Published:** 2026-09-16

**Authors:** Jing Lin, Hui-Ling Hu

**Affiliations:** 1 School of Marxism, Xiamen Ocean Vocational College, Xiamen, China; 2 Institute of Creative Design and Management, National Taipei University of Business, Taoyuan, Taiwan

**Keywords:** altruistic motivation, collectivism orientation, ideological and political education identification, PLS-SEM, social empathy, social responsibility, university students

## Abstract

This study investigates the mechanisms through which ideological and political education identification influences university students' social responsibility. Drawing on value internalization theory and prosocial behavior perspectives, this research proposes a conceptual model in which ideological and political education identification affects social responsibility through the mediating roles of social empathy and altruistic motivation, with collectivism orientation acting as a moderating variable. A quantitative survey was conducted among undergraduate students in Mainland China, and a total of 530 valid responses were collected. The data were analyzed using partial least squares structural equation modeling (PLS-SEM). The results indicate that ideological and political education identification has a significant positive effect on social responsibility. Social empathy and altruistic motivation both play significant mediating roles in this relationship. In addition, collectivism orientation negatively moderates the relationship between altruistic motivation and social responsibility. These findings suggest that the development of social responsibility among university students is shaped by a combination of value identification, emotional processes, motivational mechanisms, and cultural context. This study contributes to the literature by proposing an integrated framework explaining how ideological identification translates into socially responsible behavior among university students. The findings also provide practical implications for higher education institutions seeking to cultivate students' civic responsibility and social engagement.

## 1 Introduction

In any society, young people do not become agents capable of assuming public responsibility merely as a direct result of educational policies or the transmission of knowledge. Rather, this transformation emerges from a deeper process of value formation. Values are not simply absorbed in the form of propositions or norms; instead, they gradually take shape, are adjusted, and ultimately internalized through the dynamic interaction among cognition, emotion, and motivation. As noted by Kelman (1958, 1961), value internalization proceeds through the reconstruction of the relationships among the self, others, and the collective, so that an idea becomes an intrinsic reason for action only once it forms part of the self-system. This theoretical account is developed in detail in Section 2.1.

In this sense, Ideological and Political Education Identification is not merely the acquisition of knowledge; it represents a deeper psychological alignment process. Contemporary political psychology suggests that young people's attitudes toward the state, society, and public issues depend on how they construct moral meanings, interpret group roles, and situate the self in relation to the collective within a pluralistic information environment (Jost et al., 2022). Therefore, if ideological and political education is to genuinely motivate youths' public engagement, its influence must transcend the cognitive level and reach the more fundamental structures of value and emotion.

From the perspective of affective and moral psychology, social empathy is not merely the simulation of others' emotions, but an inner responsiveness to the lived conditions of others. Empathy opens the boundaries of the self and generates moral concern when individuals are confronted with others' vulnerability or public predicaments (Batson, 2011). Recent empirical studies further reinforce this view. For instance, Jiang et al. (2021) found in a college student sample that empathy enhances moral elevation, which in turn promotes prosocial behavior. Similarly, Peng et al. (2024) demonstrated that empathy fosters more stable altruistic tendencies among young people through the pathways of moral identity and perceived security. Collectively, these studies highlight a crucial insight: without emotional openness, values cannot be internalized, and responsibility cannot emerge. In other words, responsibility is not merely a logical conclusion of ideas—it is an extension of emotion.

However, emotion alone is insufficient to translate into action; it must be guided by motivation to develop into a sustained behavioral tendency. Altruistic motivation can be understood as the directionalization of emotion, providing empathy with an ethical purpose that enables individuals to bear personal costs and orient their actions toward the welfare of others (Fehr and Fischbacher, 2003). A growing body of recent research supports the motivational nature of altruistic behavior. For example, Wang et al. (2023) found that college students' altruistic actions often stem from moral sensibility and a sense of obligation toward others. Likewise, Cui et al. (2023) reported that altruistic motivation is rooted in emotional responsiveness while simultaneously shaped by values and cultural factors such as family functioning, indicating its dual psychological–cultural nature.

These psychological processes are never independent of cultural context. Cultural psychology posits that the self is a culturally shaped structure of perception and action (Markus and Kitayama, 2010). Young people's understanding of “what I ought to do” and “what is worth my commitment” is deeply influenced by their cultural orientations. Within a cultural framework characterized by strong collectivist orientation, individuals tend to interpret responsibility as a relational obligation rather than a matter of personal choice; the welfare of others is viewed as interconnected with the self, leading prosocial actions to carry stronger senses of duty and shared responsibility. Cross-cultural empirical evidence further indicates that cultural orientation profoundly shapes prosocial tendencies and their psychological foundations (Chen, 2024; Chui et al., 2025). Conversely, in more individualistic cultural frameworks, young people may regard value internalization primarily as an extension of personal feeling rather than an expression of collective obligation; in such a psychological configuration, empathy does not necessarily lead to responsibility, and altruistic motivation is less likely to be transformed into concrete public actions. Therefore, whether young people develop a stable sense of social responsibility cannot be judged solely by the effectiveness of educational interventions. Instead, it requires further reflection on whether they can locate a meaningful psychological position within their cultural frame that enables them to assume responsibility.

In sum, this study constructs an integrated theoretical framework in which Ideological and Political Education Identification functions as the institutional trigger for value internalization; social empathy and altruistic motivation constitute the core psychological pathways through which ideas are transformed into a tendency toward responsible action; and collectivism orientation serves as the cultural condition that strengthens or weakens this psychological process. Through this framework, the present study aims to elevate the discussion of ideological and political education to the theoretical level of how values are internalized and how responsibility is generated. By examining the interplay among values, emotions, motivation, and culture, this study seeks to uncover the deeper psychological structures and cultural logics that shape the development of social responsibility among young people.

## 2 Literature review

### 2.1 Theoretical foundations of value internalization: from attitude change to youth value formation

The development of young people's sense of public responsibility is not the direct outcome of knowledge transmission; rather, it stems from the internalization of social and political values. One of the most influential theoretical accounts of value internalization comes from Kelman (1958, 1961), who proposed three modes of attitude change: compliance, identification, and internalization. Among these, *internalization* emphasizes the transformation of external values into an individual's own belief system, enabling particular ideas to serve as intrinsic reasons for action rather than externally imposed norms. This framework highlights that values can guide behavior only when they are processed through multi-level transformations involving the self, others, and the collective, rather than remaining at the level of knowledge acquisition or superficial endorsement.

Contemporary political psychology has further advanced the understanding of value internalization by arguing that values are not static beliefs, but cognitive–motivational structures shaped through social interaction, information exposure, and group positioning. Jost et al. (2022) contend that when young people engage with public and societal issues, their attitudes result from processes of meaning-making and motivational regulation. External values can be internalized into enduring behavioral tendencies only when they resonate with an individual's self-concept and group identity.

Within this framework, Ideological and Political Education Identification is no longer viewed merely as an educational outcome but as a psychological condition that facilitates value internalization. When political and social values are accepted and integrated into an individual's self-structure, they naturally foster deeper emotional and moral transformations. Recent studies also indicate that value identification is closely linked to socio-emotional capacities (e.g., empathy) and moral behavioral tendencies (e.g., altruism) (Chen, 2024), suggesting that value internalization activates the moral–emotional system, triggering concern for others, a sense of social obligation, and stronger public responsibility.

Based on this understanding, Ideological and Political Education Identification can be regarded as a critical threshold for youth value internalization: once social and political values become incorporated into one's self-concept, the individual's perception of “others” and the “public world” is transformed. On one hand, internalized values guide young people to interpret others' circumstances from a communal perspective, thereby increasing their emotional sensitivity and empathetic capacity toward societal others (Zaki, 2020). Accordingly, this study proposes:

**H1: Ideological and Political Education Identification has a positive effect on social empathy**.

On the other hand, when values become part of the self, *assuming responsibility for society* is no longer experienced as an external requirement but as an intrinsic moral obligation, thereby reinforcing individuals' subjective sense of duty and willingness to engage in socially responsible behavior (Berkowitz and Lutterman, 1968; Hardy and Carlo, 2011). Therefore, this study posits:

**H2: Ideological and Political Education Identification has a positive effect on social responsibility**.

Moreover, value internalization reshapes the motivational structure of action, making young people more inclined to direct their behaviors toward the welfare of others and the collective, reflected in higher levels of altruistic motivation (Fehr and Fischbacher, 2003; Sortheix and Schwartz, 2017). Thus, this study proposes:

**H3: Ideological and Political Education Identification has a positive effect on altruistic motivation**.

### 2.2 Social empathy: the activation mechanism of ethical emotions and the affective foundation of public responsibility

Social empathy is a crucial psychological capacity that enables young people to engage with the public world. Its essence is not merely emotional simulation but a form of ethical sensitivity directed toward the conditions of others. As Batson (2011) argues, empathy is not only emotional resonance but also a moral response to others' needs and vulnerabilities, enabling individuals to emotionally recognize “*how I ought to respond to the other.”* This inner responsiveness forms the emotional foundation of moral action, transforming public issues from mere cognitive judgments into felt ethical calls. Research in neuroscience and moral psychology further demonstrates that empathy involves the coordinated operation of emotional responsiveness, perspective-taking, and moral reasoning systems (Decety and Cowell, 2014). Zaki (2020) adds that empathy possesses significant social functionality, promoting cooperation, enhancing group cohesion, and strengthening individuals' affective commitment to social norms.

At the behavioral level, empirical studies indicate that college students' empathy is translated into helping, cooperation, and public responsibility through the mechanism of moral identity (Peng et al., 2024), suggesting that empathy serves as a key psychological bridge between values and action. Moreover, under conditions of stress or social challenge, empathy strengthens young people's motivation to step forward (Grignoli et al., 2022), enabling them to assume roles associated with social solidarity. Within the framework of value internalization, empathy does not arise spontaneously but is instead shaped by overarching value structures. Chen (2024) found that when individuals strongly identify with particular values, their emotional systems adjust toward a communal orientation, making empathy more likely to transform into moral sensitivity and an affective sense of social obligation.

Therefore, Ideological and Political Education Identification not only influences how young people *understand* what is right, just, and public, but also shapes *how they emotionally relate to others*. Empathy thus serves as a pivotal emotional node through which value internalization evolves into public responsibility. Social empathy enhances young people's ethical engagement and sense of responsibility in public issues (Decety and Cowell, 2014; Jiang et al., 2021). Consequently, this study proposes:

**H4: Social empathy has a positive effect on social responsibility**.

Furthermore, since value internalization reshapes young people's emotional orientation toward others, social empathy naturally becomes a key mediating variable linking Ideological and Political Education Identification with social responsibility (Grignoli et al., 2022; Jiang et al., 2021). When internalized values enable young people to more deeply feel and understand others' circumstances, public responsibility is more likely to be experienced as a moral obligation rather than an external norm. Therefore, this study posits:

**H5: Social empathy mediates the relationship between Ideological and Political Education Identification and social responsibility**.

### 2.3 The formation of altruistic motivation: emotional directionality and moral action tendencies

If social empathy represents young people's ethical sensitivity toward others, then altruistic motivation is the psychological force that *directs* this ethical sensitivity toward an intention to act. Altruism is not merely a tendency to help; it is a moral driver that incorporates the welfare of others into one's own evaluative framework. Fehr and Fischbacher (2003) argue that altruistic motivation initiates action because it reshapes the value structure through which individuals assess behavioral choices—leading them to consider the interests of others and the collective when judging whether an action is “worth the effort.” Thus, altruistic motivation serves as a transitional mechanism between emotion and behavior, enabling moral sentiments to become actionable tendencies.

Moral psychology highlights that the core of altruistic motivation lies in the activation of moral identity, which links emotion to self-concept and, in turn, drives prosocial behavior. Research by Leng et al. (2020) shows that empathy strengthens college students' tendency to internalize moral traits—such as helping and caring for others—as part of their self-concept, and that the enhancement of moral identity subsequently increases prosocial behaviors such as helping and cooperation. This implies that altruism is not merely an emotional response but also a means of maintaining self-consistency—when acting for others becomes part of one's self-definition, altruistic behavior transforms into a necessary expression of self-worth. Recent findings further indicate that moral identity plays a critical bridging role between emotion and action among young people, making empathy more likely to translate into stable and sustained prosocial tendencies (Peng et al., 2024).

Within the process of value internalization, Ideological and Political Education Identification constitutes an important higher-level source for the development of altruistic motivation. When young people internalize values related to society, the nation, or the public sphere as part of their self-system, they are psychologically more inclined toward a communal orientation, and thus more likely to base their actions on the welfare of others. Once values are internalized, altruism is no longer an abstract ethical ideal but a form of self-realization (Hardy and Carlo, 2011; Leng et al., 2020). Ideological and political education identification therefore not only enhances moral knowledge but restructures young people's motivational systems in the public domain, making their actions more other-oriented and collective in nature.

Based on the above, this study proposes:

**H6: Altruistic motivation has a positive effect on social responsibility**.

Furthermore, because altruistic motivation is a key psychological channel through which value internalization translates into public-oriented behavioral tendencies, values that become concrete through the link between moral identity and other-oriented emotion are more likely to be experienced as part of the self (Hardy and Carlo, 2011; Peng et al., 2024). Consequently, young people are more inclined to perceive social obligations as integral to their personal identity. Therefore, this study posits:

**H7: Altruistic motivation mediates the relationship between Ideological and Political Education Identification and social responsibility**.

### 2.4 The psychological formation of social responsibility: from value to action

Social responsibility is not the product of external norms, nor is it an extension of knowledge retention; rather, it is a relational positioning of the self–other–public world. The emergence of a sense of responsibility depends on how individuals understand their place within the social context and derive ethical attitudes from this relational understanding. As Schwartz's theory of values suggests, values are not static beliefs but core structures through which individuals position themselves in the world (Sortheix and Schwartz, 2017). Therefore, the formation of responsibility must be rooted in the deeper processes of value internalization.

Psychological research also emphasizes that responsibility is not the outcome of pure rational calculation but the result of the joint contribution of emotional and motivational systems. Batson (2011) argues that empathy transforms others' needs into emotional burdens for the self, turning public issues into felt moral calls. Fehr and Fischbacher (2003) further contend that altruistic motivation provides the driving force for action, enabling individuals to bear costs and orient themselves toward others. Recent empirical research has shown that empathy and altruistic motivation jointly—or even interactively—predict youths' responsible actions in volunteer work, public initiatives, and civic participation (Leng et al., 2020; Peng et al., 2024). In other words, the emotional basis of social responsibility originates from empathy, while its action orientation arises from altruistic motivation.

However, the psychological formation of social responsibility among young people is not a single-layered process but one jointly shaped by educational socialization, moral–emotional development, and cultural–psychological frameworks. Educational research notes that higher education shapes not only knowledge structures but also how young people interpret the roles of the nation, society, and the self (Arthur, 2024). Developmental psychology highlights that youth is a period marked by rapid growth in moral identity and responsibility awareness; changes in emotional maturity and self-concept significantly influence the development of responsibility (Hardy and Carlo, 2011). Cultural psychology emphasizes that cultures attribute different psychological meanings to “responsibility”: collectivist cultures tend to regard responsibility as a relational obligation, whereas individualistic cultures view responsibility more as a matter of personal choice or value expression (Chen, 2024; Markus and Kitayama, 2010).

Thus, whether young people possess a sense of responsibility depends on whether they can locate a psychological position within their value, emotional, and cultural frameworks from which they can assume and respond to obligations (Markus and Kitayama, 2010). This position is not granted solely through education; rather, it emerges through a dynamic process involving:

Value Internalization → Emotional Activation → Motivational Regulation → Responsible Action

This dynamic also clarifies the logic underlying H1–H7:

• Ideological and Political Education Identification, as a higher-order value framework, enhances youths' emotional sensitivity toward others (H1), directly strengthens their responsibility cognition (H2), and reshapes their motivational structure, making altruism more intrinsic (H3).

• Empathy increases young people's ethical sensitivity toward the public world, thereby enhancing responsibility awareness (H4), and functions as the emotional pathway linking value internalization to responsible behavior (H5).

• Altruistic motivation provides the direction and energy for action, enabling responsibility to be not only “felt” but also “enacted” (H6), and deepens the effect of value internalization on responsibility formation (H7).

Under this integrative framework, social responsibility is no longer understood as a static moral attitude but as an outcome shaped by the interplay among values, emotions, and motivations—making responsibility a psychological phenomenon that is repeatedly generated within cultural contexts.

### 2.5 A cultural–psychological perspective: collectivism as a cultural condition for the formation of responsibility

A central proposition in cultural psychology is that culture is not merely an external backdrop to behavior but the very psychological architecture that shapes individuals' perceptions, emotions, and motivations. As Markus and Kitayama (2010) emphasize, culture permeates the structure of the self, shaping how people understand what constitutes the “self,” the “other,” and what is considered “the appropriate way to act.” Thus, a sense of responsibility does not emerge in a vacuum; it is embedded within the meaning frameworks that culture provides for interpreting self–other relations.

In collectivist cultures, the self is not viewed as an isolated entity but as fundamentally embedded within a network of relationships. Responsibility within this context is seen as a relational obligation—not arising from personal choice but from interdependence with family, groups, and society. Responding to others and assuming social duties are not external prescriptions but internal needs for maintaining relationships and group harmony. Within this cultural structure, moral actions carry a pronounced other-oriented quality (Markus and Kitayama, 2010; Triandis, 2001).

Recent cross-cultural research offers compelling evidence for this pattern. Chen (2024), analyzing data across multiple countries, found that cultural differences significantly shape the expression of prosocial behavior: in cultures high in collectivist orientation, young people are more inclined to view helping, cooperating, and assuming obligations as expressions of self–group consistency, rather than personal preference. In other words, culture redefines “what empathy means” and “why altruistic behavior matters,” imbuing prosocial conduct with greater moral obligation in collectivist contexts.

More specific research on Chinese youth similarly demonstrates that collectivist cultural characteristics heighten emotional sensitivity toward others and amplify social obligation. Chui et al. (2025), studying young people in Chinese cultural contexts, found that prosocial behavior is often endowed with a stronger sense of duty and other-orientation in relationship-oriented cultural environments—highlighting the critical role of cultural values in shaping youths' psychological structure for “acting on behalf of others.”

Within this cultural–psychological framework, this study proposes a key proposition: collectivism strengthens the efficiency with which empathy and altruistic motivation translate into social responsibility.

First, when empathy occurs within a collectivist cultural context, its ethical significance is magnified. Empathy becomes not merely “understanding another's emotion” but “feeling connected to the other.” When culture communicates that individuals and others belong to the same relational network, empathy is more likely to evolve into the sense that “I ought to take responsibility for the other” (Chen, 2024; Chui et al., 2025). Therefore, this study posits:

**H8: Collectivism orientation strengthens the positive effect of social empathy on social responsibility**.

Second, in collectivist cultures, altruistic motivation is more likely to be endowed with a sense of obligation. When “helping others” is understood as a moral responsibility for maintaining group cohesion, altruism is no longer an optional tendency but a core expression of self–group consistency. Therefore, when young people hold stronger collectivist orientations, their altruistic motivation is more likely to develop into stable and duty-driven public actions (Cui et al., 2023; Triandis, 2001). Based on this reasoning, the study proposes:

**H9: Collectivism orientation strengthens the positive effect of altruistic motivation on social responsibility**.

Based on the theoretical arguments and hypotheses developed in the preceding sections, this study proposes a conceptual research model to examine the mechanisms through which ideological and political education identification influences university students' social responsibility. Specifically, ideological and political education identification is proposed as the key antecedent variable, which directly influences social responsibility and indirectly affects it through the mediating roles of social empathy and altruistic motivation. In addition, collectivism orientation is incorporated as a moderating variable to examine whether cultural value orientation alters the relationships between psychological mechanisms and social responsibility. The proposed conceptual framework integrates value internalization theory, empathy–altruism theory, and cultural psychology perspectives to provide a comprehensive explanation of how educational values are transformed into socially responsible behavior among university students. The overall research model of this study is presented in Figure 1.

**Figure 1:**
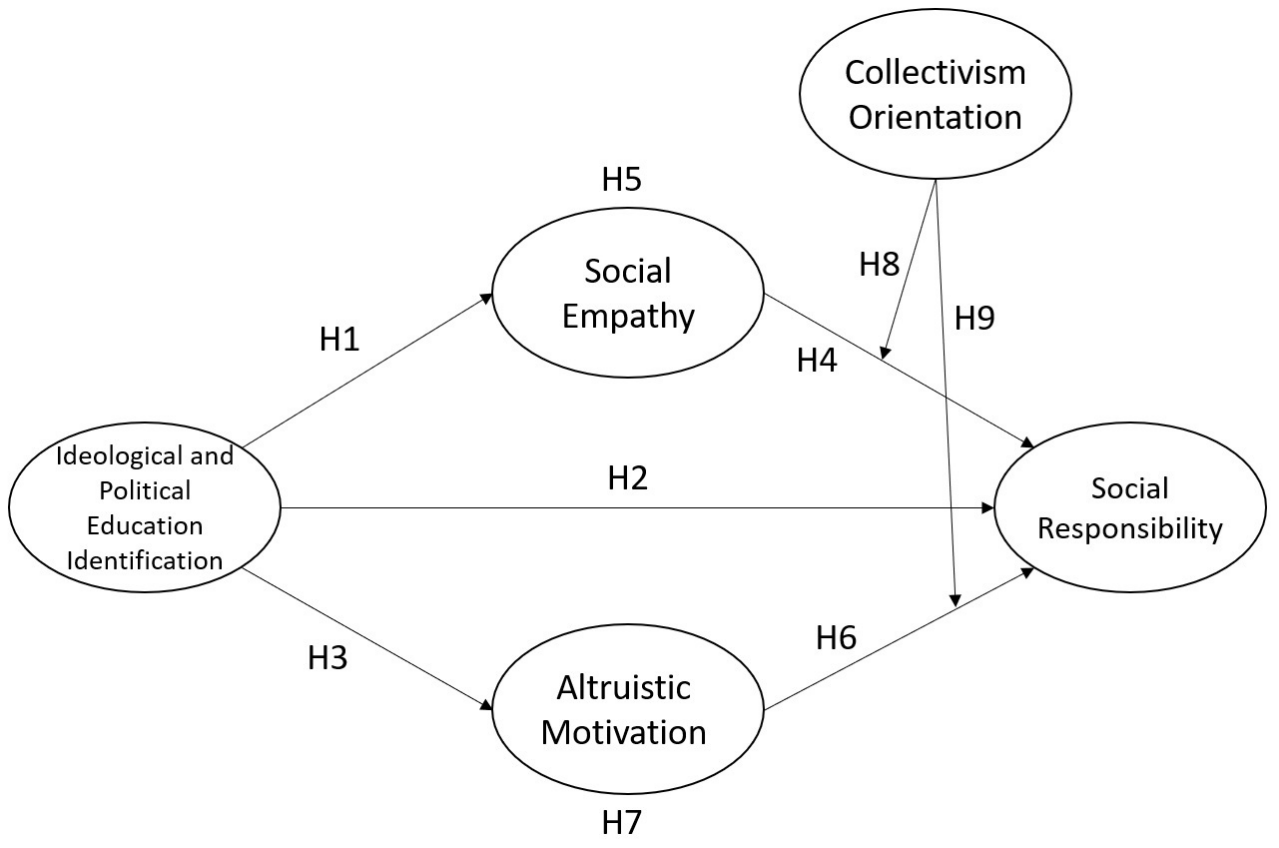
Research model.

## 3 Research method

### 3.1 Research participants and data collection

The participants of this study were undergraduate students currently enrolled in universities across Mainland China. A quantitative survey method was employed, and data were collected through an online questionnaire using convenience sampling to enhance regional coverage. The survey link was distributed through faculty members and student associations from various types of universities, including comprehensive universities, normal universities, universities of technology, and local undergraduate institutions. In addition, the questionnaire was disseminated via major social media platforms such as WeChat, QQ, and Weibo to reach students from multiple regions of China (Eastern, Central, Western, and Northeastern). Data were collected between January 1 and February 1, 2026, resulting in 530 valid responses. According to the sample size recommendations for PLS-SEM, the minimum sample size should satisfy the “10-times rule” and typically exceed 300 cases for models with complex structures such as mediation and moderation (Hair Junior et al., 2014; Hair et al., 2019). Therefore, the sample size obtained in this study is adequate to support subsequent PLS-SEM analysis.

### 3.2 Questionnaire design

This study adopted a structured questionnaire as the primary instrument for data collection. The questionnaire was developed based on the proposed research framework and consisted of two major parts: respondents' demographic information and measurement items for each research construct. Demographic variables were included to capture respondents' background characteristics and to support descriptive statistics as well as subsequent group comparison analyses. These variables included gender, year of study, academic major category, volunteer service experience, home region (Eastern, Central, Western, Northeastern China), and whether the respondent held a student leadership position. All construct items were adapted from established and validated measurement scales and were semantically adjusted to fit the context of this study, thereby enhancing relevance and comprehensibility for participants. All items were rated on a seven-point Likert scale (1 = strongly disagree; 7 = strongly agree) to measure participants' degree of agreement with each statement.

#### 3.2.1 Ideological–political education identification

The construct Ideological–Political Education Identification in this study consists of three subdimensions: cognitive identification, value identification, and affective identification. The item development primarily drew upon Kelman (1958) three-process model of social influence (compliance–identification–internalization), as well as the core concepts underlying Mael and Ashforth (1992) Organizational Identification Scale. Based on the theoretical implications of these models, measurement items were developed and further adjusted to align with the ideological–political education context of university students in Mainland China. Each subdimension contains four items, ensuring comprehensive construct coverage and adequate reliability. In total, 12 items were used to measure Ideological–Political Education Identification.

#### 3.2.2 Social empathy

The construct Social Empathy in this study was primarily measured using items adapted from the Interpersonal Reactivity Index (IRI) developed by Davis (1983). The IRI is one of the most influential and widely used SSCI-indexed instruments for empathy research and has been extensively applied in studies related to youth development, education, social behavior, and public responsibility. Specifically, this study utilized items from the Empathic Concern subscale and revised their semantic content to reflect university students' emotional responses to social issues and group-level situations in the Mainland China context. Four items were initially administered; one was subsequently removed during measurement-model purification because its outer loading fell below the recommended threshold of 0.70 (Hair et al., 2019). A total of three items were therefore retained to assess social empathy, all with outer loadings above 0.92.

#### 3.2.3 Altruistic motivation

The construct Altruistic Motivation was measured using items adapted from the Self-Report Altruism Scale (SRA) developed by Rushton et al. (1981). As a classic measure in social psychology, the SRA has been widely applied in research on youth development, volunteer behavior, prosocial tendencies, and social responsibility. Its strong theoretical and empirical grounding makes it appropriate for evaluating university students' helping motivation and prosocial inclinations in this study. A total of four items were included.

#### 3.2.4 Social responsibility

The construct Social Responsibility was measured based on the theoretical framework of the Social Responsibility Scale originally developed by Berkowitz and Lutterman (1968). This scale is a seminal instrument in social psychology for assessing public responsibility, sense of social obligation, and prosocial attitudes, and has been widely applied in research on youth moral development and civic responsibility. Drawing on the conceptual foundation of the original scale, the items were semantically adapted to reflect contemporary university students' attitudes toward public interests, social obligations, and collective welfare in Mainland China. A total of four items were used.

#### 3.2.5 Collectivism orientation

The construct **Collectivism Orientation** was measured using items adapted from Triandis (2001) influential work on cultural value orientations, particularly the Individualism–Collectivism framework. Triandis is a foundational scholar in cross-cultural psychology, and his scale has been widely applied in studies of social psychology, education, organizational behavior, and youth value identification. Based on the core principles of Triandis's model, and taking into account the value characteristics of contemporary youth, this study modified the item wording to better fit the ideological–political education context of Mainland Chinese university students. A total of four items were included.

### 3.3 Data analysis

This study employed Partial Least Squares Structural Equation Modeling (PLS-SEM) as the primary analytical method, using SmartPLS 4.0 to conduct statistical estimations. Prior to model testing, SPSS was used for data cleaning and descriptive statistical analyses to summarize the demographic characteristics of the sample. The analysis proceeded in two major stages: measurement model assessment and structural model assessment. First, the measurement model was evaluated by examining indicator reliability, internal consistency reliability (Cronbach's α and Composite Reliability), convergent validity (Average Variance Extracted, AVE), and discriminant validity using the Fornell–Larcker criterion and HTMT ratio. Only after confirming that all constructs demonstrated adequate measurement quality did the study proceed to structural model analysis. Next, the structural model was assessed. Overall model fit was first evaluated using the Goodness-of-Fit (GOF) index to ensure the robustness of the research model. Subsequently, the path coefficients, coefficient of determination (*R*^2^), and effect sizes were examined. A 5,000-sample bootstrapping procedure was used to test the significance of each structural path. Mediation effects were assessed by examining the indirect paths to determine whether social empathy and altruistic motivation mediated the relationship between Ideological–Political Education Identification and social responsibility. Moderation analysis was conducted by creating and testing interaction terms to evaluate whether collectivism orientation moderated the effects of social empathy and altruistic motivation on social responsibility.

## 4 Research results

### 4.1 Demographic characteristics of respondents

A total of 530 valid questionnaires were collected in this study. The demographic characteristics of the respondents are presented in Table 1. Female respondents accounted for 64.0% (*n* = 339) of the sample, while 36.0% (*n* = 191) were male. In terms of academic year, first-year students constituted the majority (61.7%, *n* = 327), followed by third-year (15.7%, *n* = 83) and second-year students (14.9%, *n* = 79), whereas fourth-year (3.2%, *n* = 17) and other categories (4.5%, *n* = 24) represented smaller proportions. Regarding academic majors, students in economics and management disciplines formed the largest group (37.9%, *n* = 201), followed by science and engineering (20.8%, *n* = 110) and humanities and social sciences (18.1%, *n* = 96), while medical and health-related majors accounted for 7.4% (*n* = 39) and other fields for 15.8% (*n* = 84). Most respondents reported having volunteer service experience (82.1%, *n* = 435), while 17.9% (*n* = 95) had none. In terms of family region, the largest group came from the eastern region (44.2%, *n* = 234), followed by the central (18.3%, *n* = 97), western (15.5%, *n* = 82), and northeastern regions (12.8%, *n* = 68), with the remaining respondents reporting other regions (9.2%, *n* = 49). Finally, 57.0% (*n* = 302) of respondents had served as student leaders, whereas 43.0% (*n* = 228) had not.

**Table 1:** Demographic characteristics of respondents (*N* = 530).

Category	Group	Frequency	Percentage
Gender	Male	191	36.0
	Female	339	64.0
Academic year	First year	327	61.7
	Second year	79	14.9
	Third year	83	15.7
	Fourth year	17	3.2
	Others	24	4.5
Major field of study	Humanities and social sciences	96	18.1
	Economics and management	201	37.9
	Science and engineering	110	20.8
	Medical and health sciences	39	7.4
	Others	84	15.8
Volunteer service experience	Yes	435	82.1
	No	95	17.9
Family residential region	Eastern region	234	44.2
	Central region	97	18.3
	Western region	82	15.5
	Northeastern region	68	12.8
	Others	49	9.2
Student leadership experience	Yes	302	57.0
	No	228	43.0

### 4.2 Convergent validity

The results in Table 2 indicate that the measurement model demonstrates satisfactory reliability and convergent validity. First, the outer loadings of all indicators range from 0.866 to 0.970, exceeding the recommended threshold of 0.70, which indicates adequate indicator reliability (Hair et al., 2019). Second, the Cronbach's alpha values range from 0.909 to 0.962, while the composite reliability (CR) values range from 0.936 to 0.973, both exceeding the recommended level of 0.70, suggesting strong internal consistency reliability (Hair et al., 2019; Nunnally and Bernstein, 1994). Finally, the average variance extracted (AVE) values range from 0.785 to 0.913, which are above the recommended threshold of 0.50, indicating adequate convergent validity (Fornell and Larcker, 1981). Overall, these results confirm that the measurement model meets the recommended criteria for indicator reliability, internal consistency reliability, and convergent validity, supporting the suitability of the measurement model for subsequent structural model analysis.

**Table 2:** Convergent validity of the measurement model.

Construct	Item	Outer loadings	Cronbach's alpha	Composite reliability	AVE
Ideological and political education identification	AI	0.943	0.952	0.969	0.913
	CI	0.953			
	VI	0.970			
Affective identification	AI1	0.919	0.923	0.945	0.812
	AI2	0.866			
	AI3	0.918			
	AI4	0.901			
Altruistic motivation	AM1	0.931	0.942	0.958	0.851
	AM2	0.952			
	AM3	0.888			
	AM4	0.918			
Cognitive identification	CI1	0.916	0.944	0.959	0.855
	CI2	0.912			
	CI3	0.929			
	CI4	0.941			
Collectivism orientation	CO1	0.874	0.909	0.936	0.785
	CO2	0.894			
	CO3	0.908			
	CO4	0.867			
Social empathy	SE1	0.922	0.928	0.954	0.874
	SE2	0.946			
	SE3	0.937			
Social responsibility	SR1	0.942	0.962	0.973	0.898
	SR2	0.962			
	SR3	0.941			
	SR4	0.946			
Value identification	VI1	0.916	0.950	0.964	0.870
	VI2	0.935			
	VI3	0.944			
	VI4	0.936			

### 4.3 Discriminant validity

Table 3 presents the results of the discriminant validity assessment using the Fornell–Larcker criterion. According to this criterion, discriminant validity is established when the square root of the average variance extracted (AVE) for each construct exceeds its correlations with other constructs (Fornell and Larcker, 1981). As shown in Table 3, the square root of AVE values for all constructs—including Altruistic Motivation, Collectivism Orientation, Ideological and Political Education Identification, Social Empathy, and Social Responsibility—are greater than their corresponding inter-construct correlation coefficients. These results indicate that each construct is empirically distinct from the others. Overall, the measurement model demonstrates satisfactory discriminant validity, confirming that the latent constructs capture conceptually different phenomena.

**Table 3:** Discriminant validity (Fornell–Larcker criterion).

Construct	AM	CO	IPEI	SE	SR
Altruistic motivation (AM)	**0.922**				
Collectivism orientation (CO)	0.512	**0.886**			
Ideological and political education identification (IPEI)	0.607	0.554	**0.956**		
Social empathy (SE)	0.475	0.463	0.562	**0.935**	
Social responsibility (SR)	0.666	0.652	0.730	0.681	**0.948**

Diagonal elements (bold) represent the square root of AVE values.

### 4.4 Model fit

This study employed SmartPLS 4.0 to examine the proposed conceptual model and test the research hypotheses. To evaluate the overall model fit, the Goodness of Fit (GOF) index proposed by Esposito Vinzi et al. (2010) was calculated. The GOF value is determined using the following formula: $GOF=\sqrt{AVE}\;\times \sqrt{R^{2}}$.

According to Vinzi et al. (2010), GOF values of 0.10, 0.25, and 0.36 represent small, medium, and large levels of model fit, respectively. In this study, the average AVE value was 0.857, and the average coefficient of determination *R*^2^ was 0.476. The calculated GOF value was therefore:

$$\begin{array}{l} \text{GOF}=\sqrt{0.857\times 0.476}=0.639 \end{array}$$

The resulting GOF value of 0.639 exceeds the recommended threshold for a strong model fit, indicating that the proposed conceptual model demonstrates a high level of overall explanatory power and predictive capability. These results suggest that the measurement and structural models collectively exhibit satisfactory global model fit.

### 4.5 Path analysis

Table 4 and Figure 2 present the results of the structural model analysis. Following the recommendations for PLS-SEM, the significance of the path coefficients was assessed using the bootstrapping procedure, based on the corresponding *t-values* and *p-values* (Hair et al., 2019). The results indicate that altruistic motivation (β = 0.244, *t* = 4.359, *p* < 0.001), collectivism orientation (β = 0.163, *t* = 3.399, *p* = 0.001), and social empathy (β = 0.322, *t* = 3.437, *p* = 0.001) all have significant positive effects on social responsibility. Among these predictors, social empathy exhibits the strongest effect, followed by altruistic motivation and collectivism orientation. The structural model explains a substantial proportion of variance in social responsibility (*R*^2^ = 0.743), indicating strong explanatory power (Hair et al., 2019). Furthermore, ideological and political education identification significantly influences both altruistic motivation (β = 0.607, *t* = 11.775, *p* < 0.001) and social empathy (β = 0.562, *t* = 9.037, *p* < 0.001), with explanatory power of *R*^2^ = 0.368 and *R*^2^ = 0.316, respectively, indicating moderate levels of explained variance (Hair et al., 2019). In addition, ideological and political education identification has a significant direct effect on social responsibility (β = 0.275, *t* = 3.272, *p* = 0.001). These findings suggest that ideological and political education identification not only exerts a direct influence on social responsibility but also affects it indirectly through psychological mechanisms such as altruistic motivation and social empathy.

**Table 4:** Structural model path analysis.

**Path relationship**	Original sample (β)	Standard deviation	*t* statistics	*p-values*	*R* ^2^	*f^2^*
Altruistic Motivation → Social Responsibility	0.244	0.056	4.359	0.000	0.743	0.131
Collectivism Orientation → Social Responsibility	0.163	0.048	3.399	0.001		0.050
Social Empathy → Social Responsibility	0.322	0.094	3.437	0.001		0.224
Ideological and Political Education Identification → Altruistic Motivation	0.607	0.052	11.775	0.000	0.368	0.583
Ideological and Political Education Identification → Social Empathy	0.562	0.062	9.037	0.000	0.316	0.462
Ideological and Political Education Identification → Social Responsibility	0.275	0.084	3.272	0.001		0.130

**Figure 2:**
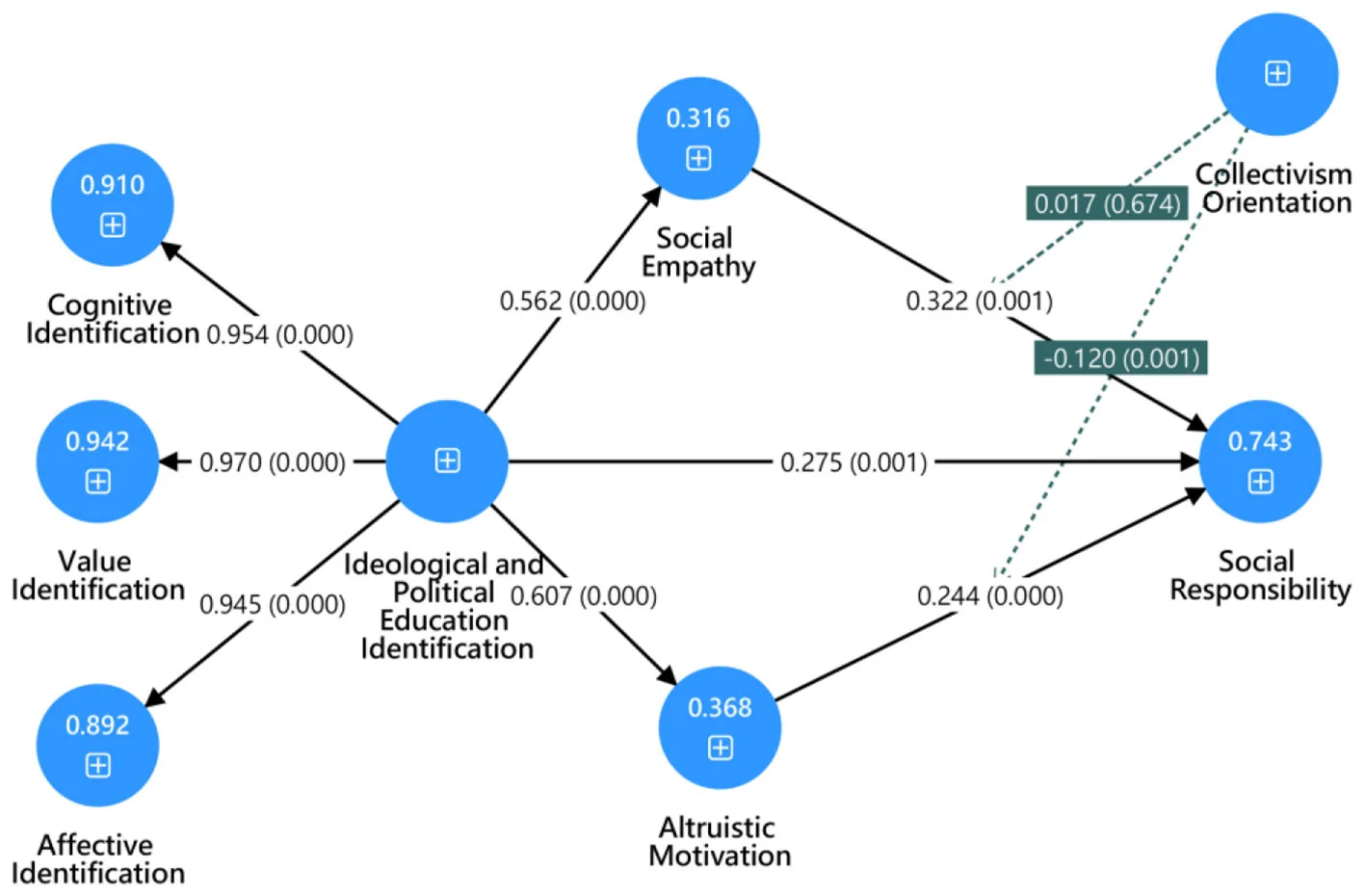
PLS-SEM statistical model diagram.

Beyond statistical significance, effect sizes (f^2^) were examined to assess the substantive contribution of each predictor. Following Cohen (1988) and Hair et al. (2019), f^2^ values of 0.02, 0.15, and 0.35 represent small, medium, and large effects, respectively. Ideological and political education identification exerted large effects on social empathy (*f*^2^ = 0.462) and altruistic motivation (*f*^2^ = 0.583), but only a small direct effect on social responsibility (*f*^2^ = 0.130). Social empathy had a medium effect on social responsibility (*f*^2^ = 0.224), whereas altruistic motivation (*f*^2^ = 0.131) and collectivism orientation (*f*^2^ = 0.050) had small effects. Thus, although the structural paths were statistically significant, their substantive magnitudes varied considerably.

### 4.6 Mediation effects

Table 5 presents the results of the mediation analysis. Following the PLS-SEM approach, the significance of indirect effects was examined using the bootstrapping procedure and the corresponding confidence intervals (Hair et al., 2019; Preacher and Hayes, 2008). The results indicate that ideological and political education identification has a significant indirect effect on social responsibility through social empathy (β = 0.181, *t* = 2.983, *p* = 0.003), with a 95% confidence interval of 0.083 to 0.315, which does not include zero. This result confirms the mediating role of social empathy. Similarly, altruistic motivation also significantly mediates the relationship between ideological and political education identification and social responsibility (β = 0.148, *t* = 3.912, *p* < 0.001), with a 95% confidence interval of 0.082 to 0.229, which also excludes zero. In addition, ideological and political education identification still shows a significant direct effect on social responsibility (β = 0.275, *p* = 0.001), indicating the presence of partial mediation. Overall, the findings suggest that ideological and political education identification influences social responsibility both directly and indirectly through the psychological mechanisms of social empathy and altruistic motivation.

**Table 5:** Mediation effect analysis.

Mediation path	Original sample (β)	Standard deviation	*t* statistics	*p-values*	2.5%	97.5%
Ideological and Political Education Identification → Social Empathy → Social Responsibility	0.181	0.061	2.983	0.003	0.083	0.315
Ideological and Political Education Identification → Altruistic Motivation → Social Responsibility	0.148	0.038	3.912	0.000	0.082	0.229

### 4.7 Moderating effects

Table 6 presents the results of the moderating effect analysis. Following the PLS-SEM approach, the significance of the interaction terms was examined using the bootstrapping procedure (Hair et al., 2019). The results indicate that collectivism orientation significantly moderates the relationship between altruistic motivation and social responsibility (β = −0.120, *t* = 3.220, *p* = 0.001). The negative interaction coefficient suggests that the positive effect of altruistic motivation on social responsibility becomes weaker as the level of collectivism orientation increases. In contrast, the interaction effect between social empathy and collectivism orientation on social responsibility is not significant (β = 0.017, *t* = 0.421, *p* = 0.674). This finding indicates that collectivism orientation does not significantly alter the relationship between social empathy and social responsibility. Overall, the results provide partial support for the proposed moderating effects, as collectivism orientation moderates only the relationship between altruistic motivation and social responsibility.

**Table 6:** Moderating effect analysis.

Path relationship	Original sample (β)	Standard deviation	*t* statistics	*p-values*	*f^2^*
Collectivism Orientation × Altruistic Motivation → Social Responsibility	−0.120	0.037	3.220	0.001	0.072
Collectivism Orientation × Social Empathy → Social Responsibility	0.017	0.039	0.421	0.674	0.002

In terms of substantive magnitude, the interaction between collectivism orientation and altruistic motivation had a small effect on social responsibility (*f*^2^ = 0.072), indicating that the statistically significant moderating effect was modest in practical terms. In contrast, the interaction between collectivism orientation and social empathy showed a negligible effect (*f*^2^ = 0.002), consistent with its non-significant path coefficient. The moderating role of collectivism orientation should therefore be interpreted with caution, particularly because the statistically significant interaction effect was small in substantive terms.

Figure 3 illustrates the moderating effect of collectivism orientation on the relationship between altruistic motivation and social responsibility. The interaction plot presents the relationship between altruistic motivation and social responsibility at three levels of collectivism orientation: one standard deviation below the mean (−1 SD), the mean level, and one standard deviation above the mean (+1 SD). As shown in the figure, altruistic motivation is positively associated with social responsibility across all levels of collectivism orientation. However, the slope of the relationship varies depending on the level of collectivism orientation. Specifically, when collectivism orientation is low (−1 SD), the positive relationship between altruistic motivation and social responsibility is stronger. In contrast, when collectivism orientation is high (+1 SD), the slope becomes flatter, indicating that the influence of altruistic motivation on social responsibility weakens as collectivism orientation increases. This pattern supports the moderating effect identified in the statistical analysis and suggests that in contexts characterized by stronger collectivist orientation, social responsibility may be driven more by social norms and collective expectations rather than by individual altruistic motivation.

**Figure 3:**
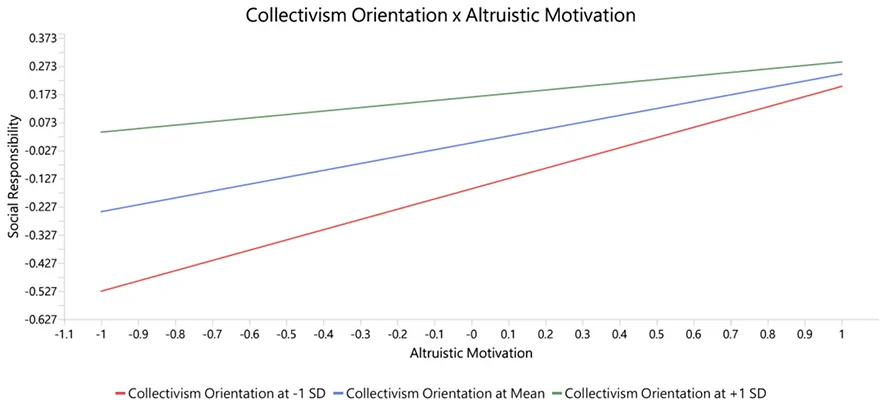
Moderating effect of collectivism orientation on the relationship between altruistic motivation and social responsibility.

## 5 Conclusions and discussion

### 5.1 Discussion of research findings

This study investigates the mechanisms through which ideological and political education identification influences university students' social responsibility, focusing on the mediating roles of social empathy and altruistic motivation and the moderating role of collectivism orientation. Using PLS-SEM analysis, the results provide empirical support for most of the proposed hypotheses and offer new insights into the psychological processes underlying the development of social responsibility among university students.

Read as a whole, the results support the integrated model rather than a series of discrete effects. Ideological and political education identification predicts social responsibility directly (β = 0.275) while also predicting social empathy (β = 0.562) and altruistic motivation (β = 0.607), and the model accounts for 74.3% of the variance in social responsibility (*R*^2^ = 0.743). The configuration of these coefficients is informative: identification functions less as a proximal determinant of responsible conduct than as an upstream value foundation that reorganizes how students feel about and orient themselves toward others. This is the sequence anticipated by value internalization theory, and it converges with two separate lines of evidence in the literature—that moral and civic education fosters empathy by widening the range of social perspectives students are willing to adopt (Decety and Cowell, 2014; Zaki, 2020), and that value-based education promotes prosocial motivation and civic engagement among young people (Leng et al., 2020). What the present data add is that the two mechanisms operate simultaneously within a single population rather than as alternative accounts.

Among the proximal predictors, the relative magnitudes are as consequential as the significance tests. Social empathy exerts the strongest effect on social responsibility (β = 0.322, *f*^2^ = 0.224, a medium effect), followed by the direct effect of identification (β = 0.275, *f*^2^ = 0.130) and by altruistic motivation (β = 0.244, *f*^2^ = 0.131), both of which are small in substantive terms, and both indirect paths are significant. The ordering carries a substantive implication: the affective route outweighs the motivational route, which suggests that what converts an endorsed value into felt obligation is primarily emotional engagement with the situation of particular others rather than a generalized disposition to benefit them. This is consistent with the empathy–altruism account of prosocial action (Batson, 2011) and with studies in which empathy predicts prosocial behavior among college students through moral rather than purely dispositional channels (Jiang et al., 2021; Peng et al., 2024), while the significant role of altruistic motivation (Fehr and Fischbacher, 2003) indicates that emotion alone does not complete the sequence. The dual mediation therefore locates the mechanism in a value–emotion–motivation sequence, and it identifies emotional resonance rather than cognitive endorsement as the point at which ideological identification becomes behaviorally consequential.

The most theoretically consequential result is the one the model did not anticipate. Collectivism orientation moderates the path from altruistic motivation to social responsibility, but the interaction is negative (β = −0.120, *p* = 0.001, *f*^2^ = 0.072)—the reverse of the direction specified in H9—whereas the parallel interaction with social empathy is non-significant and negligible in magnitude (β = 0.017, *p* = 0.674, *f*^2^ = 0.002), so that H8 is not supported. Two readings are available. The first is substitutive: where collectivist orientation is strong, socially responsible conduct is already prescribed by role obligations, group expectations and normative pressure, leaving individual altruistic motivation less incremental work to do, since the behavior occurs whether or not the student is personally oriented toward others' welfare. This is congruent with the argument that action in interdependent cultural settings is regulated by shared norms and relational duties rather than by individual dispositions (Markus and Kitayama, 2010; Triandis, 2001), and with evidence that the psychological basis of prosociality varies systematically across cultural contexts (Chen, 2024; Chui et al., 2025). The second reading is that duty-based and motivation-based responsibility constitute distinct routes to the same behavior which compete rather than accumulate; the insensitivity of the empathy path to collectivism is consistent with this, in that emotional responsiveness to a concrete other appears to operate below the level at which cultural norms substitute for personal motivation. On either reading, the result qualifies the common assumption that collectivism uniformly amplifies prosocial tendencies, and indicates that collectivism is better modeled as a boundary condition that reallocates the psychological basis of responsibility than as a general enhancer of it.

Overall, the findings of this study reveal that the formation of social responsibility among university students is shaped by a complex interplay of value identification, emotional processes, motivational mechanisms, and cultural context. Ideological and political education identification provides the value foundation, social empathy serves as an emotional driver, altruistic motivation functions as a motivational force, and collectivism orientation shapes the contextual conditions under which these mechanisms operate.

### 5.2 Theoretical implications

The present study contributes to the literature in several important ways by advancing the theoretical understanding of how ideological and political education influences the development of social responsibility among university students.

First, this study extends existing research on ideological and political education by moving beyond traditional evaluations of educational effectiveness and examining the psychological mechanisms underlying value internalization. Previous studies in this field have largely focused on institutional arrangements, curriculum design, or educational outcomes, while paying relatively limited attention to the internal psychological processes through which educational values are transformed into behavioral orientations. The present study specifies those processes empirically, showing that ideological and political education identification operates as a foundational value orientation whose behavioral consequences are carried by identifiable psychological pathways rather than by educational exposure as such. By empirically linking ideological identification to prosocial outcomes, the present study provides a more nuanced explanation of how educational values become embedded in individuals' moral and behavioral systems.

Second, this study contributes to the literature on prosocial behavior and moral psychology by highlighting the joint roles of emotional and motivational mechanisms in translating value identification into responsible social behavior. Specifically, the findings reveal that social empathy and altruistic motivation serve as complementary mediating mechanisms that connect ideological identification with social responsibility. This suggests that value-based education does not influence behavior solely through cognitive acceptance but also through emotional resonance and motivational activation. In doing so, this research integrates perspectives from empathy theory (Batson, 2011) and altruism research (Fehr and Fischbacher, 2003), proposing a “value–emotion–motivation” pathway that helps explain how moral values are transformed into socially responsible actions. The novel element, however, is not the coexistence of the two mediators but their relative weighting: the affective path proved the stronger of the two, which implies that models treating moral motivation as the principal translator of values into action may misplace the decisive step in the sequence.

Third, this study contributes to cultural psychology and value-behavior research by demonstrating that cultural orientations may shape the pathways through which individual motivations influence social responsibility. The moderating effect of collectivism orientation suggests that the motivational basis of social responsibility may differ across cultural contexts. In highly collectivistic environments, social responsibility behaviors may be guided more strongly by social norms, collective expectations, and role obligations, rather than by purely individual-level altruistic motivations. This finding provides empirical support for cultural theories suggesting that behavioral motivations are embedded within broader cultural frameworks (Markus and Kitayama, 2010). By incorporating collectivism orientation as a contextual moderator, this study advances existing models of prosocial behavior by emphasizing the importance of cultural conditions in shaping moral action.

Finally, the moderation result identifies a boundary condition that current models of prosocial behavior do not accommodate. Because collectivism weakened rather than strengthened the motivational path, cultural orientation cannot be treated as a variable that simply raises or lowers the level of prosocial output; it appears instead to govern which psychological system carries responsibility in the first place. The implication for theory is twofold: value-internalization models require an explicit specification of the cultural conditions under which individual motivation remains explanatorily necessary, and findings on the motivational basis of responsibility obtained in individualistic samples should not be assumed to transfer to interdependent settings without re-examination.

Overall, this study provides a more theoretically grounded explanation of how ideological and political education can influence students' social responsibility, highlighting the importance of psychological and cultural mechanisms in shaping socially responsible behavior.

### 5.3 Practical implications

The findings of this study provide several important practical implications for higher education institutions, educators, and policymakers seeking to enhance university students' sense of social responsibility.

First, the results suggest that universities should place greater emphasis on fostering students' value identification with ideological and political education, rather than focusing solely on knowledge transmission. Traditional ideological education often prioritizes theoretical instruction and policy interpretation. However, the findings of this study indicate that the effectiveness of such education depends largely on whether students internalize the underlying social values. Therefore, universities should adopt teaching strategies that encourage students to actively engage with social issues and reflect on the relevance of ideological values to real-life situations. For example, incorporating case-based discussions, public issue debates, and social problem analysis into ideological and political education courses may help students connect abstract ideological concepts with concrete social realities. Such approaches may strengthen students' identification with the values promoted in ideological education and ultimately enhance their sense of social responsibility.

Second, the findings highlight the critical role of social empathy in shaping socially responsible behavior among university students. Since social empathy was found to be the strongest predictor of social responsibility in this study, universities should consider integrating empathy development into their educational practices. Educational programs such as service-learning, community engagement projects, and volunteer activities can provide students with opportunities to interact with diverse social groups and gain a deeper understanding of social inequalities and societal challenges. Through these experiential learning activities, students may develop greater emotional awareness and compassion toward others, which in turn can strengthen their commitment to socially responsible actions.

Third, this study demonstrates that altruistic motivation is an important driver of social responsibility. Therefore, higher education institutions should create environments that encourage students to participate in activities that benefit society. Universities may promote altruistic values by supporting student-led volunteer organizations, establishing community service programs, and recognizing students' contributions to public welfare activities. Such initiatives can cultivate a campus culture that values cooperation, mutual assistance, and collective wellbeing, thereby reinforcing students' motivations to contribute to society.

Fourth, the moderating effect of collectivism orientation suggests that cultural values should be taken into consideration when designing educational strategies aimed at promoting social responsibility. In collectivistic cultural contexts, social behavior may be influenced not only by individual motivations but also by social norms and collective expectations. Therefore, educational institutions may emphasize the importance of collective responsibility, social harmony, and community participation when promoting social responsibility among students. By highlighting the interdependence between individuals and society, educators may encourage students to perceive socially responsible behavior as part of their social roles and obligations.

Finally, policymakers and university administrators should recognize that the development of social responsibility among university students is a multidimensional process involving value orientation, emotional engagement, and motivational factors. Educational policies aimed at strengthening social responsibility should therefore adopt a holistic approach that integrates value education, emotional development, and experiential learning. By creating supportive institutional environments that encourage civic engagement and social participation, universities can play a crucial role in cultivating socially responsible citizens capable of contributing to sustainable social development.

### 5.4 Limitations and future research

Although this study provides valuable insights into the relationship between ideological and political education identification and university students' social responsibility, several limitations should be acknowledged. These limitations also suggest potential directions for future research.

First, this study adopts a cross-sectional research design, which may limit the ability to fully capture the dynamic relationships among the variables over time. Although structural equation modeling was employed to examine the hypothesized relationships, cross-sectional data cannot fully establish causal relationships between constructs. Future studies may adopt longitudinal research designs to track changes in students' value identification, emotional responses, and socially responsible behaviors over time. Such approaches may provide a more comprehensive understanding of how social responsibility develops among university students throughout their educational experiences.

Second, the data in this study were collected using self-reported questionnaires, which may introduce the potential risk of common method bias. Although several procedural remedies were implemented in the questionnaire design, such as ensuring anonymity and using established measurement scales, responses may still be influenced by social desirability or respondents' subjective perceptions. Future research may address this issue by employing multi-source or multi-method data collection strategies, such as combining survey data with behavioral observations, peer evaluations, or participation records in social activities. These approaches may enhance the reliability and external validity of the research findings.

Third, this study focuses on university students in Mainland China, and the findings may therefore be influenced by specific cultural and social contexts. In collectivistic cultural environments, individuals' socially responsible behaviors may be shaped by social norms, collective expectations, and role obligations. As a result, the relationships observed in this study may differ in societies with different cultural orientations. Future studies may conduct cross-cultural comparative research to examine whether the proposed theoretical model operates similarly across different cultural contexts. Such research may contribute to a more comprehensive understanding of how cultural values influence the formation of social responsibility.

In addition, although this study examines the roles of ideological identification, social empathy, altruistic motivation, and collectivism orientation, the development of social responsibility among young people may be influenced by a broader range of factors. Variables such as family background, civic engagement experiences, media exposure, and personality traits may also play important roles in shaping socially responsible behavior. Future research may incorporate these additional variables to develop a more comprehensive model explaining the formation of social responsibility among university students.

Finally, this study adopts a quantitative research approach to examine the structural relationships among variables. While quantitative methods are effective in testing theoretical models, they may provide limited insights into individuals' subjective experiences and interpretations of ideological education. Future studies may employ qualitative research methods, such as in-depth interviews or focus group discussions, to explore how students interpret ideological education and how these interpretations influence their sense of social responsibility. Combining qualitative and quantitative approaches may provide a richer and more comprehensive understanding of the psychological processes underlying the development of social responsibility.

A further limitation concerns statistical inference. With 530 observations, the bootstrapping procedure affords sufficient power for comparatively small path coefficients to reach conventional significance thresholds, so statistical significance alone is a weak criterion of substantive importance in a model of this size (Hair et al., 2019). For this reason the present study reports effect sizes (f^2^) alongside the significance tests and interprets the coefficients in terms of their practical relevance. Several significant structural relationships in fact proved small in substantive terms, including the direct effects on social responsibility of identification (*f*^2^ = 0.130), altruistic motivation (*f*^2^ = 0.131) and collectivism orientation (*f*^2^ = 0.050), as did the significant moderating effect (*f*^2^ = 0.072). The smaller effects in the model, and in particular the interaction term, should nevertheless be regarded as provisional until they are replicated in independent samples, and future research is encouraged to evaluate them against explicit effect-size and predictive-relevance criteria rather than *p-values* alone.

Overall, future research may further advance this field by adopting diverse research designs, incorporating broader contextual variables, and conducting cross-cultural investigations. Such efforts may contribute to a deeper understanding of how educational values, psychological mechanisms, and cultural contexts jointly shape the development of social responsibility among university students.

## Data Availability

The datasets analyzed for this study can be found in the Zenodo data repository at https://doi.org/10.5281/zenodo.19180839.
